# Psychosocial effects of an Ebola outbreak at individual, community and international levels

**DOI:** 10.2471/BLT.15.158543

**Published:** 2016-01-21

**Authors:** Tine Van Bortel, Anoma Basnayake, Fatou Wurie, Musu Jambai, Alimamy Sultan Koroma, Andrew T Muana, Katrina Hann, Julian Eaton, Steven Martin, Laura B Nellums

**Affiliations:** a Cambridge Institute of Public Health, University of Cambridge, Box 113, Cambridge Biomedical Campus, Forvie Site, Robinson Way, Cambridge, CB2 0SR, England.; b United Nations Mission for Ebola Emergency Response, Freetown, Sierra Leone.; c Canada Grand Challenges Project, ADDO/Caritas Freetown, Freetown, Sierra Leone.; d Social Mobilization Action Consortium, Freetown, Sierra Leone.; e Ministry of Health and Sanitation, Freetown, Sierra Leone.; f International Organization for Migration, Freetown, Sierra Leone.; g CBM International, Lomé, Togo.

## Abstract

The 2013–2016 Ebola outbreak in Guinea, Liberia and Sierra Leone was the worst in history with over 28 000 cases and 11 000 deaths. Here we examine the psychosocial consequences of the epidemic. Ebola is a traumatic illness both in terms of symptom severity and mortality rates. Those affected are likely to experience psychological effects due to the traumatic course of the infection, fear of death and experience of witnessing others dying. Survivors can also experience psychosocial consequences due to feelings of shame or guilt (e.g. from transmitting infection to others) and stigmatization or blame from their communities. At the community level, a cyclical pattern of fear occurs, with a loss of trust in health services and stigma, resulting in disruptions of community interactions and community break down. Health systems in affected countries were severely disrupted and overstretched by the outbreak and their capacities were significantly reduced as almost 900 health-care workers were infected with Ebola and more than 500 died. The outbreak resulted in an increased need for health services, reduced quality of life and economic productivity and social system break down. It is essential that the global response to the outbreak considers both acute and long-term psychosocial needs of individuals and communities. Response efforts should involve communities to address psychosocial need, to rebuild health systems and trust and to limit stigma. The severity of this epidemic and its long-lasting repercussions should spur investment in and development of health systems.

## Résumé

La flambée de maladie à virus Ebola qui a frappé la Guinée, le Libéria et la Sierra Leone en 2013-2016 a été la pire de toute l'histoire, avec plus de 28 000 cas et 11 000 décès. Dans ce dossier, nous examinons les conséquences psychosociales de cette flambée épidémique. La maladie à virus Ebola est une maladie traumatisante, compte tenu à la fois de la gravité de ses symptômes et des taux de mortalité qui y sont associés. Les personnes affectées sont susceptibles de développer des troubles psychologiques à cause de l'évolution traumatisante de l'infection, de la peur de mourir et du fait de voir d'autres personnes mourir autour d'eux. Les survivants peuvent aussi avoir des séquelles psychologiques liées à un sentiment de honte ou de culpabilité (pour avoir transmis l'infection à d'autres personnes, par exemple) ou à cause de leur stigmatisation ou de leur mise en accusation au sein de leur communauté. À l'échelle communautaire, un schéma cyclique de peur intervient, avec une perte de confiance envers les services de santé, et la stigmatisation des personnes affectées entraîne une rupture des interactions au sein de la communauté et une fracture de la communauté. Dans les pays touchés, les systèmes de santé ont été lourdement ébranlés et même dépassés par la flambée de la maladie. Leur capacité s'est considérablement réduite, près de 900 agents de santé ont été infectés et plus de 500 sont décédés. Cette flambée épidémique a majoré les besoins de services de santé, réduit la qualité de vie et la productivité économique et entraîné la fracture du système social. La réponse internationale doit absolument tenir compte aussi bien des besoins psychosociaux immédiats des individus et des communautés que de ceux à plus long terme. Il serait judicieux que les communautés soient intégrées dans les efforts de riposte pour répondre aux besoins psychosociaux, reconstruire les systèmes de santé, rétablir la confiance des populations et limiter les stigmatisations. La gravité de cette flambée épidémique ainsi que ses répercussions durables devraient inciter à investir dans les systèmes de santé et à les consolider.

## Resumen

El brote de Ebola en 2013 y 2016 en Guinea, Liberia y Sierra Leona fue el peor de la historia, con más de 28 000 casos y 11 000 muertes. En este artículo se examinan las consecuencias psicosociales de la epidemia. El Ebola es una enfermedad traumática, tanto por la gravedad de sus síntomas como por las tasas de mortalidad. Los afectados pueden sufrir efectos psicológicos dado el proceso traumático de la infección, temer a la muerte y ser testigos de la muerte de otros. Los supervivientes también pueden sufrir consecuencias psicosociales debido a los sentimientos de vergüenza y culpa (por ejemplo, por transmitir la infección a otros) y la estigmatización o reproche de sus comunidades. A nivel comunitario, se produce un patrón cíclico de temor que se traduce en la pérdida de la confianza en los servicios sanitarios, lo que da lugar a la interrupción de las interacciones de las comunidades y una ruptura de las mismas. Los sistemas sanitarios de los países afectados se vieron gravemente perjudicados y desbordados por el brote, y sus capacidades se redujeron significativamente, puesto que casi 900 trabajadores sanitarios fueron infectados por el virus del Ebola y más de 500 murieron. El brote provocó una mayor necesidad de servicios de salud, redujo la calidad de vida y la productividad económica y fracturó el sistema social. Es fundamental que la respuesta mundial al brote tenga en cuenta las profundas necesidades psicosociales a largo plazo, tanto para individuos como para comunidades. Las medidas de respuesta deberían comportar que las comunidades abordasen las necesidades psicosociales, reconstruyesen los sistemas sanitarios y la confianza y redujesen la estigmatización. La gravedad de esta epidemia y sus repercusiones a largo plazo deberían estimular la inversión y el desarrollo de los sistemas sanitarios.

## ملخص

لقد كانت عدوى حمى الإيبولا في عامي 2016 و2013 في غينيا وليبيريا وسيراليون هي الأسوأ في التاريخ من خلال حدوث أكثر من 28,000 حالة إصابة بالمرض و11,000 وفاة. ونحن نقوم هنا بفحص ودراسة الآثار النفسية والاجتماعية للوباء. الإيبولا هي مرض رضحي من حيث شدة الأعراض ومعدلات الوفيات على حد سواء. ومن المحتمل أن يتعرض المصابون لتأثيرات نفسية واجتماعية نظرًا لسير العدوى على نحو رضحي، والخوف من الموت، والتعرض لتجربة مشاهدة الآخرين يموتون في كل مكان حولهم. كما يمكن أن يتعرض الناجون أيضًا لآثار نفسية واجتماعية بسبب مشاعر الخجل أو الشعور بالذنب (على سبيل المثال نقل العدوى للآخرين) والإحساس بالوصم أو اللوم من مجتمعاتهم. على المستوى المجتمعي، يحدث نمط الخوف الدوري مع فقدان الثقة في الخدمات الصحية والحرج الاجتماعي، التي بدورها تؤدي إلى اختلال تفاعلات المجتمع وانقسام المجتمع. لقد تعثرت الأنظمة الصحية في البلدان المتضررة بشدة وأرهقت بسبب حدوث العدوى وانخفضت قدراتها بشكل ملحوظ حيث أصيب ما يقرب من 900 عامل في مجال الرعاية الصحية بعدوى الإيبولا وتوفي أكثر من 500 عامل. وأدى تفشي العدوى إلى ازدياد الحاجة للخدمات الصحية، وانخفاض نوعية الحياة والإنتاجية الاقتصادية واختلال النظام الاجتماعي. ومن الضروري أن تقوم الاستجابة العالمية للعدوى بمراعاة الاحتياجات النفسية والاجتماعية الحادة وطويلة الأجل للأفراد والمجتمعات على حد سواء. وينبغي أن تشمل جهود الاستجابة المجتمعات لمعالجة الحاجة النفسية والاجتماعية، ولإعادة بناء الأنظمة الصحية واستعادة الثقة وتقييد الحرج الاجتماعي. إن شدة هذا الوباء وتداعياته المستمرة على الأجل البعيد أمور من شأنها تشجيع الاستثمار في الأنظمة الصحية وتحسينها.

## 不只是传播病毒：埃博拉疫情对感染个人和社区的心理影响摘要

2013-2016 年在几内亚、利比里亚和塞拉利昂爆发的埃博拉疫情是史上最严重的一次，共导致了 28 000 例病患和 11 000 例死亡。我们研究了疫情对心理产生的后果。就其症状严重性和死亡率而言，埃博拉病毒是一种创伤性疾病。那些被感染的患者可能会由于感染所造成的创伤、对死亡的恐惧和见证其他人死亡的经历而饱受心理影响。幸存者还可能由于羞愧内疚（例如传染给其他人）和承受来自其所在社区的谴责或责备而饱受心理苦果。在社区水平上，会周期性出现恐惧现象，同时伴随着对医疗服务丧失信心以及羞辱感，从而导致社区干预的瓦解和社区功能的破坏。由于有约 900 名医疗护理工作者感染埃博拉病毒，其中 500 多人已死亡，所以受影响国家的医疗系统遭到本次疫情的严重破坏并且不堪重负，从而导致其能力也大幅度降低。本次疫情导致对医疗服务的需求有所增加、生活品质有所降低，同时经济生产力和社会系统遭到破坏。对本次疫情的全球响应既要考虑个人和社区的短期心理需求，也要考虑其长期心理需求。响应工作应让社区参与进来以解决心理需求，从而在重建医疗系统和重塑信任感的同时，限制羞耻感的萌生。该疫情的严重程度及其长期影响应该刺激对医疗系统的投资以及医疗系统的发展。

## Резюме

Вспышка вирусной лихорадки Эбола в Гвинее, Либерии и Сьерра-Леоне в 2013–2016 годах была самой тяжелой в истории: заболело около 28 000 человек, из них 11 000 умерло. В данном документе анализируются психологические последствия этой эпидемии. Лихорадка Эбола — травмирующее заболевание как по степени тяжести симптомов, так и в связи с высоким уровнем смертности. Те, кто с ним сталкиваются, испытывают, вероятнее всего, психологическое воздействие в связи с очень тяжелым протеканием инфекции, страхом смерти и в связи с тем, что они наблюдают, как погибают другие люди. Те, кто выжили после болезни, могут также испытывать чувство стыда или вины в качестве психологических последствий (например, из-за того, что от них заразились другие люди), а также могут стать жертвами обвинений и стигматизации со стороны своей общины. На уровне общин существует волнообразное нарастание и снижение страха смерти в сочетании со стигматизацией больных и потерей доверия к органам здравоохранения, что приводит к нарушению связей внутри общины и к ее распаду на более мелкие группы. Системы здравоохранения в странах, ставших жертвой эпидемии, сильно пострадали: они испытывали огромное напряжение в связи со вспышкой инфекции, при этом их возможности были сильно снижены, так как почти 900 медицинских работников были инфицированы вирусом Эбола и более 500 из них умерли. В результате вспышки заболевания выросла потребность в услугах здравоохранения, ухудшилось качество жизни и экономическая производительность общества, а сообщества оказались раздробленными и разрушенными. Крайне важно, чтобы реакция мирового сообщества на вспышку этого заболевания учитывала как острые, так и долговременные психологические потребности пострадавших лиц и сообществ в целом. Усилия, направленные на ликвидацию последствий эпидемии, должны включать привлечение сообществ к удовлетворению психологических потребностей их членов, к восстановлению системы здравоохранения и доверия к ней, а также к ограничению стигматизации больных. Тяжесть эпидемии и ее долговременные последствия должны привлечь инвестиции в системы здравоохранения и способствовать их дальнейшему развитию.

## Introduction

The 2013–2016 Ebola virus disease epidemic was the largest ever recorded with over 28 000 cases and 11 000 deaths.^1^ Guinea, Liberia and Sierra Leone experienced the most widespread transmission. It is essential that the global community is not complacent as continued efforts are needed to ensure that the virus transmission is controlled in the short term and that longer-term consequences of the epidemic are addressed.

A person infected with Ebola virus has well recognized signs and symptoms of fever, headache, joint and muscle pain, widespread bleeding, diarrhoea and other physical symptoms leading to high mortality. An Ebola epidemic does not only affect physical health, but also has psychosocial implications at individual, community and international levels, both acutely and in the long term (Table 1). Here we examine the psychosocial consequences of Ebola on these three levels.

**Table 1:** Acute and long-term psychosocial effects of an Ebola epidemic at individual, community and international levels

Level	Acute effects	Long-term effects
Individual	Fear and/or anxiety (e.g. of infection, death, separation from or loss of loved ones) Shame and/or guilt Frustration, anger or helplessness Stigma and/or isolation Grief and/or loss	Trauma (e.g. from course of infection, witnessing death of others) Grief and/or loss Mental health problems
Community	Fear and/or anxiety Stigma and/or isolation Grief and/or loss Disruption to community and cultural life	Loss of trust (e.g. in health services) Community fracturing Grief and/or loss Loss of support or coping resources
International	Fear and/or anxiety (e.g. of infection) Trauma (e.g. of international aid workers witnessing deaths caused by Ebola virus) Stigma and discrimination Loss of economic investment, business, travel and tourism	Trauma and long-term mental health problems (e.g. of international aid workers witnessing deaths caused by Ebola virus) Stigma and discrimination Loss of economic investment, business, travel and tourism

## Individual level

To understand the psychosocial effects of the Ebola epidemic at the individual level, we considered three groups: survivors, contacts and carers.^2^

### Survivors

Ebola is a traumatic illness both in terms of symptom severity and mortality rates. Those affected are likely to experience psychological effects due to the traumatic course of the infection, fear of death and experience of witnessing others dying.^3^ Survivors can also experience psychosocial consequences due to feelings of shame or guilt (e.g. from transmitting infection to others) and stigmatization or blame from their communities.^3^^–^^5^ Some survivors were threatened, attacked, evicted, left behind by, or excluded from, their families and communities because they were seen as tainted and dangerous. Fear and stigma of Ebola are contributed to by cultural beliefs (e.g. being a bewitched disease with those affected at fault or deserving their illness),^6^^,^^7^ widespread fears due to high infection risk, lack of information and misinformation.

### Contacts

Contacts of those infected with Ebola also experience stigmatization and isolation.^3^^,^^6^ Witnessing the traumatic course of the infection in others can result in fear and anxiety about falling ill or dying themselves, in addition to feelings of loss and grief from losing loved ones.^5^

Since Ebola is transmitted through contact with bodily fluids, loved ones are often separated from the sick upon showing symptoms and are unable to be with them as they suffer or die. This can increase feelings of grief, loss or distress and feelings of guilt or helplessness for being unable to comfort or care for loved ones.^5^ In the authors’ experiences, quarantining protocols to reduce Ebola transmissions also led to stigma and community isolation.

Since the infection can be transmitted after death, traditional mourning practices, which involve cleaning and touching dead bodies while preparing them for burial, are very risky. In many cases, bodies of the deceased are removed and buried by trained burial teams to prevent transmission, which might compound the loss experienced by loved ones, preventing traditional rites or coping processes for grieving, paying respects or gaining closure. These disruptions to traditional practices can result in feelings of resentment, anger or fear (e.g. beliefs about misfortune when not paying respect to the deceased) and can reduce access to community support usually associated with traditional mourning practices. Loss of support resources further limits ability to cope and increases distress.^5^^,^^8^

### Carers

Those treating the sick (e.g. community or family carers, traditional healers and health workers) can also experience psychological effects. Witnessing the traumatic course of the infection and their patients’ death puts carers at risk of poor psychological outcomes, including anxiety, depression and post-traumatic stress disorder.^2^^,^^3^^,^^9^ Often, those assuming carer responsibilities are family members, particularly young people, who can experience increased anxiety, frustration and grief because of their relationship with the patient. Given the high fatality rate and lack of treatment for Ebola, carers can feel burdened by guilt for being unable to adequately look after or save the patients. Working long hours, overwhelming patient numbers, limited safety equipment and a feeling of inability to provide adequate care for, or heal, those infected can also result in frustration, anger or feelings of helplessness for health workers.^2^^,^^3^

Because of the severity of symptoms and high mortality rates, Ebola carers can feel significant fear, anxiety or helplessness in relation to their own risk of infection and death. Carers have been reported to experience psychosomatic Ebola symptoms.^2^^,^^3^^,^^8^^,^^9^ Carers can also experience isolation from their families or community because of the infection transmission risk (e.g. through elective or mandatory quarantine) and stigmatization due to fear or mistrust.^8^ For instance, there were myths or rumours that Ebola is administered by health workers or that it is a plot by foreign governments.^10^ In some cases, health-care workers and even their families have been evicted, threatened or attacked,^3^^,^^8^ including eight health-care workers who were killed in Guinea while raising awareness about Ebola.^11^

## Community level

At the community level, a cyclical pattern of fear occurs, with a loss of trust in health services and stigma, resulting in disruptions of community interactions and community fracturing.^3^^–^^5^^,^^7^^,^^8^ A communal sense of grief can also be felt due to significant loss of community members. This can have further psychosocial consequences as communities face shifting roles to adapt to the loss of parents, breadwinners, carers, teachers and community leaders.^4^ Communities also face structural repercussions (e.g. disruptions to business and industry, closure of community services, markets and schools, decreased health and support services), which might result in longer-term psychosocial effects.^4^ Health systems in affected countries were severely disrupted and overstretched by the outbreak and their capacities were significantly reduced as almost 900 health-care workers were infected with Ebola and more than 500 died.^1^

## International level

The 2013–2016 Ebola outbreak also had international psychosocial implications. Reports of stigma, discrimination and blame targeted at communities perceived to be of African descent in other non-African countries increased due to fear of infection.^12^^–^^14^ The fear contributed to a decline in interactions with countries with widespread transmission, including the provision of resources (e.g. health workers), economic investment, business, industry, travel and tourism. Health workers returning from affected countries experienced stigmatization too.^12^^,^^15^

The psychosocial effects of the Ebola outbreak reflect those of other emergencies, particularly epidemics, where communities and health workers are exposed to disease and psychosocial stressors, exacerbating existing challenges. Responses to this type of emergency should be informed by guidelines for identifying and responding to psychosocial effects of global health crises.^16^ Furthermore, facilitators and barriers for addressing psychosocial effects of this epidemic should be documented, with reports or reviews of previous responses to crises, to strengthen international response mechanisms.

## Discussion

The Ebola outbreak has profound psychosocial implications at individual, community and international levels. Response efforts should involve communities to address psychosocial needs, to rebuild health systems and trust, and to limit stigma.^7^

Integrating local knowledge and understanding of illness with biomedical approaches to achieve culturally relevant, acceptable and appropriate psychosocial interventions is essential.^6^^,^^7^ Strategies include communication, education, community engagement, peer support, resource mobilization and prevention activities (e.g. risk assessment, psychosocial support) as well as mental health care. Interventions and policy initiatives should embrace survivor engagement to solicit and learn from their experiences, to influence policy and practice, including efforts to address physical, psychological and social care needs (e.g. stigma and re-integration).

It is important that the discourse and implementation of strategies addressing needs resulting from the epidemic should not further marginalize or stigmatize affected communities. Such marginalization is compounded by a focus on factors exacerbating the epidemic and weaknesses in these communities, rather than their strengths (e.g. social resources or resilience), which not only disempowers them but also inhibits success by failing to integrate existing resources.

We should also recognize existing efforts that address psychosocial effects of the outbreak, for example mental health training for health workers, psychological first aid and significant support from within communities and the global community such as donations, volunteers, community mobilization, peer support and awareness-raising.^2^^,^^5^ However, significant needs remain in addressing psychosocial care after the Ebola outbreak, as requested by many survivors. Psychosocial care has been insufficient to date due to a lack of resources, overburdened health systems and a lack of knowledge about supporting psychosocial needs.

A multi-faceted approach is needed to address the psychosocial consequences of this epidemic at individual, community and international levels. The psychosocial effects of the outbreak, and subsequent lessons learnt, should not only inform acute responses to Ebola, but also the development of health systems and strategies to respond to future Ebola epidemics. Lessons learnt from previous responses to such crises by the global community and regulations (both beneficial and ineffective strategies) should be revisited in forming responses to this Ebola epidemic. Initiatives providing psychosocial support should integrate and strengthen traditional, social and psychological support structures, while recognizing resilience and drawing on positive empowering resources existing within communities.

## Conclusion

Due to a sustained lack of investment in health systems,^17^^,^^18^ communities in developing countries are vulnerable to both outbreaks and their psychosocial repercussions, which compound health needs. In the case of Ebola, there was a failure to respond, both by the global and local communities, to recognized risks of an outbreak that were clearly identified by the global community during previous epidemics.^16^ The severity of this epidemic and its long-lasting repercussions should spur investment in and development of health systems, including for mental and physical health. While there is now investment dedicated to rebuilding health systems in West Africa, it is essential that the global response to Ebola considers psychosocial needs and is committed to robust community-based initiatives so that health systems will be better prepared in future.
